# Mitotic chromosomes harbor cell type– and species-specific structural features within a universal loop array conformation

**DOI:** 10.1101/gr.280648.125

**Published:** 2025-08

**Authors:** Marlies E. Oomen, A. Nicole Fox, Inma Gonzalez, Amandine Molliex, Thaleia Papadopoulou, Pablo Navarro, Job Dekker

**Affiliations:** 1 Department of Systems Biology, University of Massachusetts Chan Medical School, Worcester, Massachusetts 01605, USA;; 2 Howard Hughes Medical Institute, Chevy Chase, Maryland 20815, USA;; 3 Department of Developmental and Stem Cell Biology, Institut Pasteur, Université Paris Cité, CNRS UMR3738, Epigenomics, Proliferation, and the Identity of Cells Unit, 75015 Paris, France

## Abstract

Mitotic chromosomes are considered to be universally folded as loop arrays across species and cell types. However, some studies suggest that features of mitotic chromosomes might be cell type– or species-specific. We previously reported that CTCF binding in human differentiated cell lines is lost in mitosis, whereas mitotic mouse embryonic stem cells (mESC) display prominent binding at a subset of CTCF sites. Here, we perform footprint ATAC-seq analyses of mESCs, and somatic mouse and human cells, confirming these findings. We then investigate roles of mitotically bookmarked CTCF in prometaphase chromosome organization by Hi-C. We do not find any remaining interphase structures, such as TADs or loops, at bookmarked CTCF sites in mESCs. This suggests that mitotic loop extruders condensin I and II are not blocked by CTCF and, thus, that maintained CTCF binding does not alter mitotic chromosome folding. Lastly, we compare mitotic Hi-C data generated in this study in mouse with public data in human and chicken. We do not find any cell type–specific differences; however, we find a difference between species. The average genomic size of mitotic loops is smaller in chicken (200–300 kb) compared to human (400–600 kb) and especially mouse (1–1.5 Mb). Interestingly, we find that this difference is correlated with the genomic length of q-arms in these species, a finding we confirm by microscopy measurements of chromosome compaction. This suggests that the dimensions of mitotic chromosomes can be modulated through control of loop size by condensins to facilitate species-appropriate shortening of chromosome arms.

The development of 3C techniques (Dekker et al. 2002; Dostie et al. 2006; Lieberman-Aiden et al. 2009; Belaghzal et al. 2017) has contributed to a better understanding of key features of chromosome organization in vertebrate cells. Interphase chromosomes are organized on the megabase scale in A and B compartments, and each can be subdivided in smaller subcompartments (Rao et al. 2014; Spracklin et al. 2023), and on a smaller scale of tens to hundreds of kilobases in topologically associating domains (TADs) (Lieberman-Aiden et al. 2009; Dixon et al. 2012; Nora et al. 2012; Rao et al. 2014; Michieletto et al. 2016; Erdel and Rippe 2018; Nuebler et al. 2018). TADs are proposed to be formed by loop extruding machines, such as cohesins, which can be blocked by the chromatin binding protein CCCTC-binding factor (CTCF) when bound to its motif (Rao et al. 2014, 2017; de Wit et al. 2015; Sanborn et al. 2015; Dekker and Mirny 2016; Fudenberg et al. 2016; Nora et al. 2017; Nuebler et al. 2018). Although the mechanisms that establish and maintain these structures are largely shared between different cell types and between different vertebrate species, the specific genomic regions that interact can differ strongly between species, cell types, and even between sick and healthy cells (Rao et al. 2014; Lupiáñez et al. 2015; Dekker and Mirny 2016; Smith et al. 2016; Valton and Dekker 2016; Oksuz et al. 2021).

In contrast to interphase chromatin, vertebrate mitotic chromosomes are often thought to all fold as arrays of loops that are sequence-independent, independent of cell type or organism, and regardless of the diversity of macroscopic shapes they can adopt (Kieserman and Heald 2011; Kubalová et al. 2023; Zhou et al. 2023; Câmara et al. 2024). Historically studied by microscopy (Flemming 1878; Marsden and Laemmli 1979; Earnshaw and Laemmli 1983) and, in more recent years, using genomics techniques (Naumova et al. 2013; Gibcus et al. 2018; Abramo et al. 2019), we have gained understanding on the fundamental principles of mitotic chromosome folding. In mitosis, the interphase structures are completely dissolved, as both TADs and compartments can no longer be observed (Naumova et al. 2013; Gibcus et al. 2018). Instead, chromosomes are folded as helical loop arrays mediated by condensin I and II, which are not positioned at any specific genomic locations (Belmont 2006; Gibcus et al. 2018; Batty and Gerlich 2019). This results in the observation of a generally smooth and genome-wide inverse relationship between genomic distance and interaction frequency without any site-specific features, when studying mitotic chromosomes in cell populations by Hi-C (Naumova et al. 2013; Gibcus et al. 2018).

This might give the impression that mitotic chromosomes in all biological contexts are organized in a similar fashion. However, microscopy and biochemical studies revealed that condensins play a more complex role during the rapid cell cycle of mouse embryonic stem cells (mESCs) (Fazzio and Panning 2010). It has been shown in *Xenopus leavis* that mitotic chromosomes from sperm nuclei are folded as long and thin structures but become increasingly shorter and fatter throughout the early stages of development (Kieserman and Heald 2011). Additionally, depletion experiments in *Xenopus leavis* extract experiments show that the ratio of condensin I and II can affect the width-to-length ratio of chromosomes in mitosis (Shintomi and Hirano 2011; Zhou et al. 2023). Along these lines, it has been described recently that the degree of chromosome arm compaction during mitosis can differ across species (Kakui et al. 2022).

Using genomics techniques, it was found that mitotic chromosomes can harbor cell type–specific features on a more detailed scale, for example, in chromatin accessibility at the level of the nucleosomal array, histone modifications, and mitotically bound chromatin factors (Wang and Higgins 2013; Hsiung et al. 2015; Festuccia et al. 2016, 2019; Oomen et al. 2019). Of particular interest are studies that found that architectural protein CTCF remains bound to a subset of its binding sites during mitosis in some cell lines, whereas it is completely displaced in others: In differentiated human cell lines HeLa, U2OS, and HFF, we have previously reported complete loss of CTCF binding by ATAC-seq, CUT&RUN, and imaging (Oomen et al. 2019). Similarly, we described complete (3T3) or nearly complete (C2C12) loss of binding in mouse somatic cell lines (Owens et al. 2019). In contrast, we showed in mESCs that a substantial fraction of CTCF sites remains bound in mitosis (Owens et al. 2019), and this persistent association has been linked to CTCF-dependent postmitotic reactivation of a small subset of promoter-restricted mitotic CTCF targets (Chervova et al. 2023). Moreover, mitotic CTCF binding was also associated with faster reassembly of 3D contacts during early interphase of pluripotent cells (Pelham-Webb et al. 2021). These observations are in line with independent observations in a mouse blood progenitor cell line, in which the retained CTCF binding has been implicated in faster transcription reactivation, when involving promoters, and more generally in fast restoration of 3D contacts after mitosis (Zhang et al. 2019). Together, these reports suggest that mitotic chromosomes are not strict universal structures across eukaryotes and that the overall dimensions of the mitotic loop array arrangement as well as the local chromatin state can reflect both species-specific features as well as characteristics of its cell type identity.

In this study, we first performed parallel footprinting analyses of ATAC-seq data to confirm that mitotic CTCF binding is prominent in mESCs only. Notably, comparative Hi-C analyses did not show any conformational specificity associated to mitotic CTCF binding, indicating that mitotically retained CTCF sites do not influence condensin-mediated loop extrusion and mitotic chromosome formation. Interestingly, these analyses revealed species-specific differences in mitotic chromatin loop sizes in relation to differences in genomic arm length. We find that mitotic chromosome folding principles are insensitive to species- and cell type–dependent differences in CTCF retention and that mitotic chromosome conformation is adaptable through modulation of loop sizes to generate chromosomes of appropriate dimensions.

## Results

### A subset of CTCF sites remains bound in mitotic mESCs

In recent years, several genomics studies have reported contradictory results on the cell cycle binding dynamics of CTCF, especially during mitosis (Oomen et al. 2019; Owens et al. 2019; Zhang et al. 2019). These studies did not only differ in the choice of cell type but also methodologically, with some cell lines being analyzed by ATAC-seq (HeLa, HFF, U2OS, and mESC [Oomen et al. 2019; Owens et al. 2019]), some by CUT&RUN (HeLa [Oomen et al. 2019]), and others by ChIP-seq (mESC, C2C12, 3T3, G1E-ER4 [Owens et al. 2019; Zhang et al. 2019]). Using ChIP-seq, ATAC-seq, and CUT&RUN, it was shown that, in human or mouse differentiated cell lines, either all CTCF sites lose binding in mitosis (Oomen et al. 2019; Owens et al. 2019; Zhang et al. 2019) or show minor signs of mitotic binding (Owens et al. 2019; Zhang et al. 2019); in contrast, ATAC-seq and ChIP-seq revealed extensive mitotic binding of CTCF in mESCs (Owens et al. 2019). It is possible that these differences are the result of the use of different methods. However, these studies do not only differ in genomics techniques and crosslinking conditions, but more notably, they differ in which cell line was used. We hypothesized that reported differences in mitotic retention of CTCF could result from a difference in cell types and species. This would suggest that pluripotent cells can maintain partial CTCF binding in mitosis, whereas somatic cell lines lose CTCF binding in mitosis. To test this directly, we compared data obtained with identical experimental methods for pluripotent and somatic cell lines: we compared previous ATAC-seq data generated in pluripotent mouse ESCs (Festuccia et al. 2019) with newly generated ATAC-seq data in differentiated mouse C2C12 cells, using footprinting analyses previously used to show the full eviction of CTCF from human somatic cells in mitosis (Oomen et al. 2019). First, we directly compared previous collections of CTCF binding sites (Owens et al. 2019) that were shown by ChIP-seq to either maintain full binding in mitosis (bookmarked; 10,799 sites), exhibit reduced but detectable binding (reduced; 18,704 sites), or display a complete loss of binding (lost; 22,302 sites) (Owens et al. 2019). By representing ATAC-seq data as V-plots (Zentner and Henikoff 2014; Oomen et al. 2019), we can not only observe accessibility but also footprints at these specific sets of CTCF sites. When CTCF is bound to chromatin, it will occupy ∼80 base pairs around its motif. Furthermore, it will push the neighboring nucleosomes away from the motif and into a well-positioned tight array on each side of the motif (Fu et al. 2008; Oomen et al. 2019; Owens et al. 2019). We can observe these phenomena when we represent ATAC-seq data of nonsynchronized mESCs aggregated around CTCF sites that are known to be bound in interphase based on ChIP-seq data (Supplemental Fig. S1A). First, the arms of the V cross at ∼80-bp fragment length, the known footprint size of CTCF (Fu et al. 2008). Second, along the arms of the V, dots of enriched signal appear at regular intervals (∼280 bp, ∼460 bp, ∼640 bp, etc.). This ATAC-seq signal indicates the array of well-positioned nucleosomes flanking the bound CTCF motif (Fu et al. 2008). Previously, we found that, in differentiated cell lines HeLa, U2OS, and HFF, CTCF sites generally lost accessibility in mitosis (Oomen et al. 2019). When ATAC-seq signal of mitotic differentiated cells was plotted as V-plots, we found that CTCF sites no longer showed enrichment at 80-bp fragment length. Instead, the fragment size dropped to much smaller fragment size, suggesting a loss of CTCF binding in mitosis in differentiated cell lines (Oomen et al. 2019).

When we created V-plots for all interphase-bound CTCF sites in both nonsynchronized (Supplemental Fig. S1A) and mitotic (Supplemental Fig. S1E) mESCs, we observed a less clear picture. First, more accessibility is maintained at CTCF sites in mitotic mESCs compared to differentiated cell lines reported previously (Oomen et al. 2019). When we performed a side-by-side comparison of V-plots of nonsynchronized and mitotic cells at the CTCF motif (Supplemental Fig. S1I), we observed that the size of the CTCF footprint and the positioning of the nucleosomes along the arms of the V drop down to shorter fragment sizes in mitosis. However, this change is less drastic than what we have observed before in differentiated cell lines. This suggests that there are CTCF sites that maintain mitotic binding as well as CTCF sites that lose binding during mitosis, as we had observed using ChIP-seq (Owens et al. 2019). Indeed, we find that mitotically bookmarked sites (Supplemental Fig. S1B,F,J) maintain both ATAC-seq signal and a prominent CTCF footprint in mitosis, indicating high occupancy binding. Additionally, we observe a stronger signal, indicating nucleosomal positioning along the arms of the V-plot. This suggests a stronger nucleosome phasing in mitotic mESCs at CTCF bound sites. We note that, in differentiated mitotic cells, we observed before that ATAC-seq reveals a stronger nucleosome repeat length pattern throughout the genome, suggesting that this is a general phenomenon for mitotic chromatin (Oomen et al. 2019). In contrast, at sites that lost CTCF binding, ATAC-seq signal decreases and the fragment size of the CTCF footprint drops to shorter fragments, confirming the loss of CTCF binding (Supplemental Fig. S1D,H,L,N). ATAC-seq signal at CTCF sites that showed reduced ChIP-seq signal in mitotic mESCs show a more ambiguous footprint when plotted as V-plots (Supplemental Fig. S1C,G,K,M). This suggests that this category contains sites that are less frequently bound, either in single cells or in the population, an observation that can be extended to lost CTCF sites, which display reduced CTCF footprints in interphase compared to bookmarked sites. Accordingly, the quality of the CTCF motif at lost sites is largely inferior to bookmarked sites (Owens et al. 2019).

**Figure 1.**
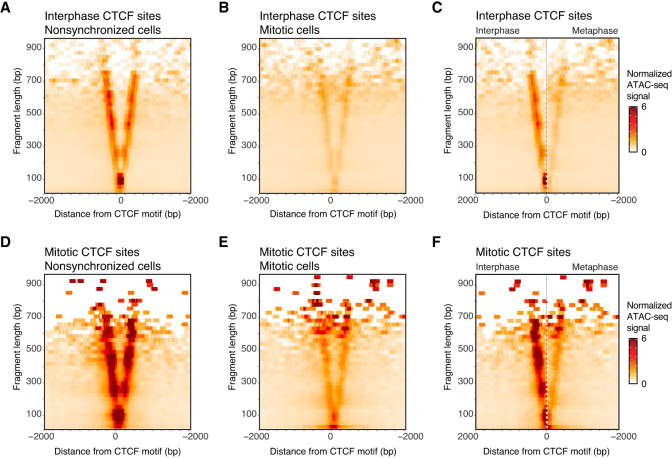
ATAC-seq data in C2C12 cells show that CTCF binding is largely lost in mitosis. (*A*–*C*) ATAC-seq data of nonsynchronized (*A*) and mitotically synchronized (*B*) C2C12 cells represented in V-plots as a pile-up on all interphase-bound CTCF sites (5827 sites total), as well as a side-by-side comparison of V-plots for nonsynchronized and mitotically synchronized cells on interphase bound CTCF sites. (*D*–*F*) ATAC-seq data of nonsynchronized (*D*) and mitotically synchronized (*E*) C2C12 cells represented in V-plots as a pile-up on all mitotic-bound CTCF sites (526 sites total), and a side-by-side comparison of the footprint of mitotically bound CTCF sites in nonsynchronized and mitotic cells (*F*).

To determine whether this partial retention of CTCF along mitotic chromosomes is seen for other mouse cell lines, we performed ATAC-seq with the differentiated mouse cell line C2C12—a cell line derived from muscle tissue (Fig. 1). We find dramatic loss of accessibility of interphase bound CTCF sites in mitosis (5827 interphase accessible CTCF sites vs. 526 in mitosis) as well as a loss of binding of CTCF to its motifs when data are represented as V-plots (Fig. 1A–C). This observation is highly similar to what we previously reported for human differentiated cell lines (Oomen et al. 2019). We note, however, that the CTCF footprint is not fully lost in mitosis, despite the clear loss of accessibility as observed by loss of signal in the V-plots as well as a reduction in the number of peaks called at CTCF sites, which is substantially lower than the number of bound CTCF motifs in mitotic mESCs as described above observed by ChIP-seq (51,805 interphase bound CTCF sites vs. 29,503 bookmarked or reduced CTCF sites in mitosis). The maintained accessibility in mitotic C2C12 cells is particularly noticeable when only visualizing mitotically accessible CTCF motifs as V-plots (Fig. 1D–F), where we see the remnants of the typical CTCF footprint at 80- to 100-bp fragment size as well as the increase of signal at the CTCF motif itself of very short fragments (<50 bp). This could be explained in two ways: (1) It is possible that a small fraction (<10%) of CTCF sites remains bound in mitosis in part of the cell population; or (2) despite efforts of cell synchronization, a small fraction of the cell population is not fully arrested in prometaphase but instead has not yet reached full prometaphase arrest or has escaped the mitotic nocodazole arrest.

Taking together these and previous results of ATAC-seq footprinting analyses (Oomen et al. 2019) confirms that the variable conclusions in the literature regarding mitotic retention of CTCF binding are in large part related to cell state differences rather than to technical and analytical differences, with pluripotent cells showing prominent bookmarking of CTCF sites, whereas differentiated cells do not.

### Loss of CTCF-related architectural features in mitosis independently of CTCF binding

**Figure 2.**
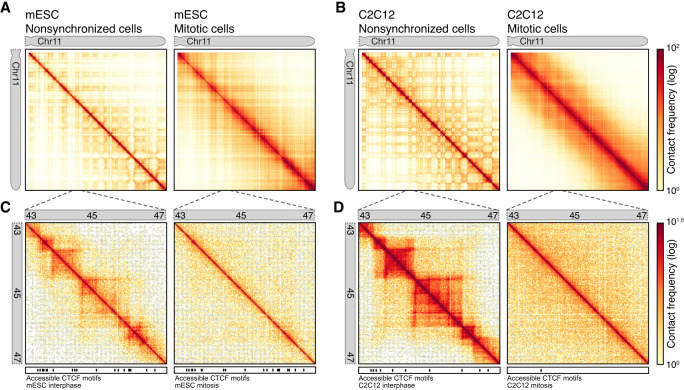
Hi-C data show compartments and TADs are lost in both mitotic mESCs and C2C12. (*A*,*B*) Hi-C heat map of Chr 11 at 100-kb bins for mESCs (*A*) and C2C12 (*B*) nonsynchronized cells (*left* panel) and mitotic arrested and sorted cells (*right* panel). (*C*,*D*) Zoom-in Hi-C heat map of Chr 11: 43,000,000–47,000,000 at 25-kb bins for mESC (*C*) and C2C12 (*D*) for nonsynchronized cells (*left* panel) and mitotic arrested and sorted cells (*right* panel).

The finding that a substantial fraction of CTCF sites maintains binding to mitotic chromosomes in mESCs raises the question whether CTCF can still function as an architectural protein in mitosis. In interphase cells, chromatin-bound CTCF can block loop extrusion mediated by cohesin (Fudenberg et al. 2016). This results in the formation of TADs and strong interactions between two CTCF sites (CTCF-CTCF loops), which are readily observed by Hi-C (Dixon et al. 2012; Nora et al. 2017; Rao et al. 2017). In mitotic differentiated cell lines, where CTCF binding is lost, no TADs and CTCF-CTCF or any other site-specific loops are observed (Naumova et al. 2013; Gibcus et al. 2018; Oomen et al. 2019). Maintained CTCF binding in mitotic mESCs creates the opportunity to study whether mitotic loop extruding machines condensin I and II can be blocked by CTCF, or whether they can shape the characteristic densely packed consecutive loop array unimpeded by bound CTCF. We performed Hi-C on nonsynchronized and mitotically synchronized mESCs (Fig. 2A). In addition to this, we also performed Hi-C on mouse nonsynchronized and mitotically sorted C2C12 cells (Fig. 2B), a differentiated cell line which largely lose CTCF binding in mitosis (Fig. 1), similar to the human differentiated cell lines previously analyzed (Oomen et al. 2019).

When we plot Hi-C data on a chromosome-wide level (Fig. 2A,B), we observe in interphase cells from both mESC and C2C12 clear compartment structures, represented as a checkerboard pattern in the heat maps. Interestingly, the compartment signal in mESCs is much less pronounced compared to C2C12 cells. The strengthening of compartment signal during differentiation has recently been described in human cell lines (Oksuz et al. 2021). However, we note that mESCs have a shorter cell cycle and lack a G1/S checkpoint and, thus, have a much higher proportion of cells undergoing replication (∼60%) than most differentiated cells (∼20%), which may affect the strength of interphase structures as observed by Hi-C (Nagano et al. 2017). When we next examine chromosome-wide heat maps of mitotic cells, we find that compartments are lost in both C2C12 cells and mESCs. This is in line with the previous observations in differentiated human cell lines, where compartment signal is lost entirely in mitosis as well (Naumova et al. 2013). We then examined a smaller 4-Mb region within Chr 11 to observe presence or absence of TADs. Whereas in nonsynchronized cells, TADs can be observed in both mESCs (Fig. 2C) and C2C12 cells (Fig. 2D), in mitosis these structures are lost.

### Mitotic loop extrusion is not blocked by retained CTCF sites

**Figure 3.**
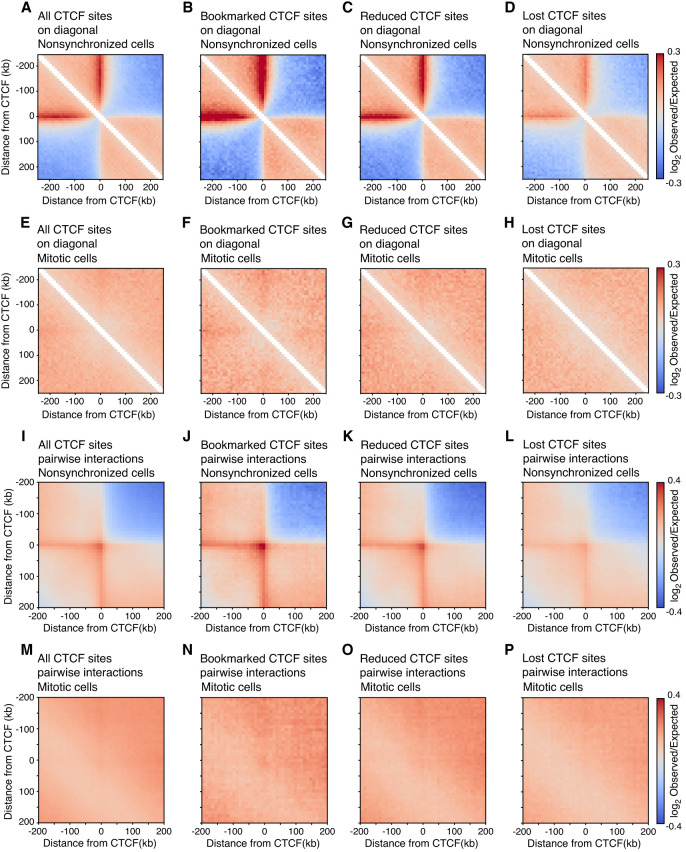
Hi-C pile-up plots on single and pairwise CTCF sites show that loop extrusion by condensins in mitosis cannot be blocked by bound CTCF. (*A*–*D*) Aggregate of Hi-C signal binned at 10 kb in nonsynchronized mESCs on all interphase-bound CTCF sites (*A*), mitotic bookmarked sites (*B*), reduced CTCF sites (*C*), and CTCF sites that lose binding in mitosis (*D*). (*E*–*H*) Aggregate of Hi-C signal in mitotic mESCs on all interphase-bound CTCF sites (*E*), mitotic bookmarked sites (*F*), reduced CTCF sites (*G*), and CTCF sites that lose binding in mitosis (*H*). (*I*–*P*) Pile-up of Hi-C signal in 10-kb bins in nonsynchronized (*I*–*L*) and mitotic (*M*–*P*) mESCs of pairwise interactions within 250 kb at all interphase bound CTCF sites (*I*,*M*), bookmarked CTCF sites (*J*,*N*), reduced CTCF sites (*K*,*O*), and CTCF sites that lose binding in mitosis (*L*,*P*). All CTCF sites are plotted with respect to strand orientation of the motif.

Next, we set out to analyze CTCF-anchored loops in mitotic mESCs to more directly investigate whether mitotic loop extruders condensin I and II are blocked by bound CTCF, which would lead to positioned loops between pairs of CTCF sites. As described above, no compartments and TADs are detected in mitotic mESCs at individual genomic locations (Fig. 2). Assessment of the presence of CTCF-dependent loops at specific locations typically requires much deeper sequencing (Akgol Oksuz et al. 2021). To observe loop formation using our mESC Hi-C data sets, loops can be visualized by plotting the aggregate Hi-C signal at and around either single CTCF sites (Fig. 3A–H) or on pairwise interactions of CTCF sites (Fig. 3I–P). In line with the above described ATAC-seq analysis, we used CTCF sites that are categorized based on published ChIP-seq data (Owens et al. 2019) in mESCs as mitotic bookmarked sites, mitotically reduced sites, and sites that lose CTCF binding in mitosis.

When we aggregate Hi-C signal at and around individual interphase-bound CTCF-sites (i.e., on the diagonal of the Hi-C interaction map), a strong insulating domain boundary can be observed at the center of the pile-up plot in interphase cells (Fig. 3A). This represents the accumulation of insulating potential of CTCF at TAD boundaries, as it reduces the interaction frequency between loci across the bound CTCF site (Dixon et al. 2012; Nora et al. 2017). Insulation can be the result of blocked loop extrusion at CTCF sites and is lost when cohesins are depleted (Rao et al. 2017). Given that blocking of extrusion depends on the orientation of the CTCF motif, a stripe of enriched interactions is detected starting at the CTCF motif and continuing in only one direction. Such directional stripes are hallmarks of blocked loop extrusion and have been reported before (Fudenberg et al. 2016; Vian et al. 2018). Strong evidence for blocked loop extrusion is observed when aggregating Hi-C interactions from nonsynchronized cells on mitotically bookmarked CTCF sites (Fig. 3B), reduced CTCF sites (Fig. 3C), and lost CTCF sites (Fig. 3D). We note that the insulation potential is strongest for bookmarked CTCF sites, compared to that observed at reduced and lost CTCF sites, in line with the differential intensity of CTCF binding at these sites and the presence of motifs of different quality (Owens et al. 2019). Similar to the ATAC-seq experiments described above, Hi-C is performed on a population of cells. Therefore, a possible explanation for the quantitative difference in insulation at these three categories of CTCF sites could be that, in interphase, bookmarked CTCF sites are more likely to be bound by CTCF across the population, whereas reduced and lost CTCF sites are also captured in unbound states in the population. In contrast, when we plot these same pile-up plots for Hi-C data obtained from mitotic mESCs, we see that all CTCF insulation is lost for each category of CTCF sites (Fig. 3E–H). This strongly implies that loop extrusion in mitosis is not blocked at sites where CTCF binding is maintained (bookmarked and reduced sites).

Likewise, we can plot the aggregation of Hi-C signal on pairwise CTCF looping interactions. We curated a list of all possible pairwise interactions between two CTCF sites separated by up to 250 kb. Typically, pairwise CTCF looping interactions are enriched in Hi-C interaction signal in interphase, as can be observed as a dot in the center of the pile-up plot representing loops between pairs of CTCF sites, combined with flanking stripes caused by the directionality of the CTCF motif and the typical forward-reverse CTCF-CTCF looping interaction (Rao et al. 2014; de Wit et al. 2015). Indeed, we see a clear enrichment at pairwise CTCF interactions in nonsynchronized mESCs across all categories of CTCF sites (Fig. 3I–L). This enrichment at pairwise CTCF sites is lost in mitosis for all three categories of CTCF sites (Fig. 3M–P). Combined, these results suggest that although CTCF binding is maintained in mitosis at a substantial fraction of sites in mESCs, CTCF does not have the ability to block mitotic loop extruders condensin I and II, and therefore no CTCF-CTCF loops are formed. These results also strongly suggest that, by prometaphase, there are no extruding cohesin complexes active on the chromosomes, as previously suggested by Smc1 ChIP-seq in nocodazole-arrested mESCs (Owens et al. 2019). In a recent study, we showed that most, if not all, extrusive cohesin is removed from chromatin during early mitosis (prophase) and that this removal is facilitated by condensins (Samejima et al. 2025).

### Mitotic loop sizes differ between species

Hi-C data can be represented as a distance decay plot, where the interaction frequency *P* is plotted as a function of the genomic distance *s*. These *P*(*s*) plots have distinct shapes for both interphase and mitotic chromosomes (Naumova et al. 2013). By calculating the slope of *P*(*s*) and plotting the derivative of contact frequency as a function of genomic distance, the average loop sizes present in interphase and mitosis can be revealed (Gassler et al. 2017; Haarhuis et al. 2017; Schwarzer et al. 2017; Gibcus et al. 2018; Abramo et al. 2019; Polovnikov et al. 2023). Such derivative plots display a characteristic local peak around 1–200 kb for interphase cells and at larger genomic distances for mitotic cells, corresponding to the genomic distance where *P* decays most slowly. This genomic distance is correlated to the average loop size, generated by either cohesins (in interphase), or condensins (in mitosis) (Gassler et al. 2017; Gibcus et al. 2018; Polovnikov et al. 2023).

**Figure 4.**
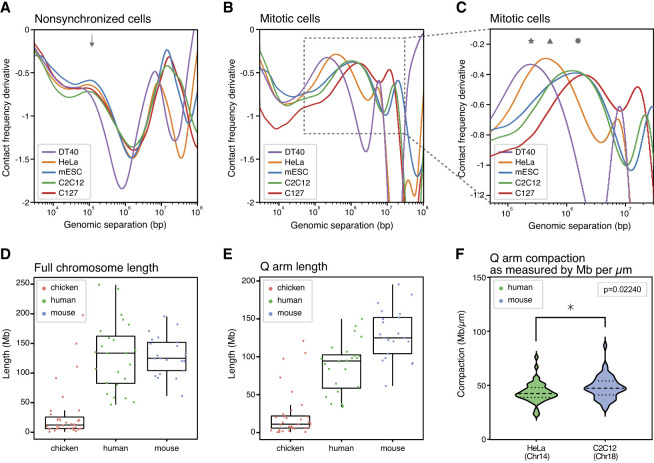
Mitotic loop arrays differ in average loop size between species. (*A*) Derivative of *P*(*s*) as a function of genomic separation in nonsynchronized chicken cells (DT40), human cells (HeLa), and mouse cells (mESCs, C2C12, and C127). The arrow highlights the average loop size mediated by cohesin in interphase in all cell types and species (*B*) Derivative plots of Hi-C data from chicken cells (DT40), human cells (HeLa), and mouse cells (mESCs, C2C12, and C127) synchronized in mitosis. (*C*) A zoom-in of the derivative plot shown in Figure 4B. The star highlights the average loop size observed in mitotic chicken cells (250 kb), the triangle highlights the average loop size in mitotic human cells (450 kb), and the circle highlights the average loop size in mitotic mouse cells (1.25 Mb). (*D*) Box plot of full chromosome lengths in chicken genome (galGal6), human genome (hg38), and mouse genome (mm10). Dots represent individual chromosomes. (*E*) Box plot of all q-arm lengths in chicken genome (galGal6), human genome (hg38), and mouse genome (mm10). Dots represent individual chromosomes. (*F*) Q-arm compaction as measured by microscopy as Mb/µM in mitotically synchronized HeLa cells (Chr 14) and C2C12 (Chr 18). Asterisk shows significant difference between arm compaction in mouse and human (n = 50, unpaired *t*-test).

In addition to any differences between stem cells and differentiated cells, we were interested to study the loop characteristics of different species in interphase and mitosis. We supplemented the Hi-C data generated in this study in mouse and human with data from several studies which included Hi-C data on both nonsynchronized and mitotic cells in different species (Abramo et al. 2019; Fitz-James et al. 2020; Samejima et al. 2025). This enabled the comparison of chicken cells (cell line DT40), human cells (cell line HeLa), and mouse cells (cell lines mESCs, C2C12, and C127). In nonsynchronized cell populations, Hi-C data from all species, and cell types behaved similarly (Fig. 4A) with an average interphase loop size of ∼100 kb (as highlighted with the arrow in Fig. 4A). Interestingly, this is not the case for mitotic loops of these different species (Fig. 4B and zoom in Fig. 4C). Although there is no difference between the estimated mitotic loop sizes as observed by the derivative plots of the three mouse cell lines analyzed (mESCs and the differentiated cell lines C2C12 and C127), a clear difference is observed between mitotic cells of human, mouse, and chicken (Gibcus et al. 2018). All mouse cell lines show an average mitotic loop size of 1–1.5 megabases (Fig. 4C, highlighted with circle), whereas human cell line HeLa shows a loop array size of 400–600 kb in mitosis (Fig. 4C, highlighted with triangle), and chicken cell line DT40 has an average loop size of 200–300 kb (Fig. 4C, highlighted with star). We note that mitotic loop sizes as determined by *P*(*s*) plots are highly consistent between replicates (Supplemental Fig. S2C) and are not strongly affected by technical conditions, such as synchronization length, hours of prometaphase arrest, or Hi-C protocol (Supplemental Fig. S2D). We note that a second peak can be observed in the derivative plots around 10 megabases. This reflects the helical organization of the mitotic loops, as was first observed in DT40 cells by Gibcus et al. (2018). Lastly, chromosomes can differ by centromere position and length, between species as well as within a given species. Here, we represent the combined data of all chromosomes to represent in our *P*(*s*) plots, but we note that we did not find differences when calculating derivative plots for different chromosomes within a given species (Supplemental Fig. S2A; note that the derivative plots of different chromosomes perfectly overlap), even when comparing acrocentric chromosomes of similar length between species (Chr 14 for both mouse and human). Combined, this suggests a different level of mitotic compaction between the three species.

We hypothesized that this difference in loop sizes could be related to the genomic lengths of chromosomes in the different species. When loops are longer, mitotic chromosomes will become shorter. Possibly, longer chromosomes require a higher level of compaction (shortening along their length), which can be achieved by formation of larger mitotic loops, to ensure proper separation of sister chromatids during anaphase. When we plot all genomic lengths of all chromosomes of the three species (Fig. 4D), it becomes clear that chicken chromosomes are, on average, much shorter than human and mouse chromosomes, with a few chromosomes being almost as long as human chromosomes. Mouse and human chromosomes have similar average chromosome length, but the longest mouse chromosome is considerably shorter than the longest human chromosome. The centromere is an important region of mitotic chromosomes where the mitotic spindle will attach, which will pull the sister chromatids apart during anaphase (McKinley and Cheeseman 2016). We realized that it is, therefore, more relevant to plot the length of the longest arm of each chromosome, per definition, the q-arm, rather than plotting the full chromosome lengths. Indeed, when we compare the q-arm length between these three species, we find that chicken has very short q-arms with an average length of 11 Mb, followed by human chromosomes with an average q-arm length of 94 Mb, and an average q-arm length of 125 Mb for mouse chromosomes (Fig. 4E). For a given organism, loop size is most likely set to ensure that the longest arms are sufficiently compacted. The longest arm in chicken cells is shorter than the longest arm in human cells, and the longest arm in human cell is shorter than the longest arm in mouse. To confirm the hypothesis that loop sizes along mitotic chromosomes are regulated to ensure appropriate shortening of chromosomes, we experimentally measured the q-arm length in mitotic human and mouse cells for two chromosomes of highly similar length by microscopy (Chr 18 in mouse and Chr 14 in human, both acrocentric chromosomes with q-arm lengths ∼90 Mb). As expected, because mitotic loops are larger in mouse (Fig. 4C; Supplemental Fig. S2B), we find that mouse Chromosome 18 compacts (shortens) to a greater extent than human Chromosome 14, reflected in a higher megabase per micrometer length ratio (Fig. 4F; Supplemental Fig. S3).

Combined, these results show that, in the cell lines we investigated, mitotic loop sizes are not related to cell type or differentiation state but instead differ among species. Moreover, our results suggest that there is a relationship between the longest genomic q-arm length and the level of mitotic chromosome compaction through genome-wide modulation of mitotic loop size, as shown using both genomics and microscopy techniques. We propose that this ensures that even the longest arms are sufficiently compacted to ensure their segregation.

## Discussion

In this study, we set out to explore mitotic chromosome organization in different cell types and vertebrate species. Although mitotic chromosomes are often perceived as universal rod-shaped structures and folded into series of compressed loops (Câmara et al. 2024), we find there are several characteristics that differ between differentiation state and between species. First, using a single analytical method in side-by-side comparisons, we confirm partial maintenance of CTCF binding in mitotic mESCs and a large eviction in differentiated cells, whether originating from mouse or human (Oomen et al. 2019; Owens et al. 2019). Interestingly, when mESCs are investigated by Hi-C, we observe that no interphase structures are maintained in mitosis despite maintained CTCF binding, suggesting that CTCF does not block mitotic loop extrusion by condensins and a loss of loop extruding cohesin complexes, as also previously suggested by SMC1 ChIP-seq data (Owens et al. 2019). Lastly, we investigate whether mitotic chromosomes are differently organized between species. For this analysis, we generated Hi-C data for mouse cell lines and analyzed publicly available data for mitotic human and chicken cell lines (Abramo et al. 2019; Fitz-James et al. 2020; Samejima et al. 2025). Although further experiments will be necessary, we find that the sizes of mitotic loops are different between species but do not change between different cell lines of the same organism. Furthermore, our results suggest that mitotic loop size, and therefore the degree of chromosome compaction, are correlated with the average length of the q-arm of chromosomes, a phenomenon that we confirmed by microscopy.

The result that mESCs maintain bookmarking of CTCF binding at a substantial fraction of sites raises the key question of why it is largely evicted in most, if not all, differentiated cell types displaying condensed chromosomes, including mouse sperm cells in meiosis II (Jung et al. 2017) and mouse oocytes (Wang et al. 2024). CTCF is a C2H2 zinc finger protein, which are canonical transcription factors subject to mitotic phosphorylation that abolishes their DNA binding capacity (Dovat et al. 2002; Dephoure et al. 2008; Rizkallah and Hurt 2009). Indeed, previous observations showed CTCF is phosphorylated during mitosis (Sekiya et al. 2017). We note that, although the different cell lines in this study warrant different synchronization protocols, the duration of the mitotic arrest by nocodazole is unlikely to cause a difference in CTCF binding or phosphorylation status. In a previous study using untreated cycling cells, we have observed the loss of stable chromatin binding of CTCF in mitotic U2OS cells by super resolution microscopy (Oomen et al. 2019). Furthermore, using a separate synchronization protocol following mitotic release after G2 arrest in chicken cells, we have found that CTCF becomes maximally dissociated as soon as cells enter prometaphase (Samejima et al. 2025). Thus, the eviction of CTCF in mitosis might be the norm and its retention in mESCs a result from a lack of phosphorylation events that merit experimental validation in the future. Alternatively, it is also possible that the chromatin remodelers associated with CTCF binding, such as SNF2H/L (Wiechens et al. 2016), may be differentially regulated in mitotic mESCs. A second important question raised by our findings is to what extent mitotic binding by CTCF could be functional. Recent work has shown that, whereas mitotic CTCF binding correlates with rapidly reactivated genes after mitosis (Owens et al. 2019; Zhang et al. 2019; Pelham-Webb et al. 2021; Chervova et al. 2023), the depletion of CTCF at the M/G1 transition affects a minor fraction of its mitotic targets and, especially, those displaying promoter restricted binding (Zhang et al. 2019; Chervova et al. 2023). Nevertheless, correlative studies have suggested that mitotic CTCF binding events are associated with early TAD restoration after mitosis (Pelham-Webb et al. 2021), and the functional depletion of CTCF during M/G1 transition was found associated with a general lack of TAD formation in G1 and the persistence of inappropriate enhancer-promoter contacts (Zhang et al. 2019). Thus, it is possible that mitotic binding events of CTCF, particularly in mESCs, are required for the fidelity of gene regulation more than for transcription levels per se. Interestingly, we note that mouse stem and progenitor cells have a much faster cell cycle compared to many differentiated cell lines (∼12 h in mESCs vs. 24 h in HeLa cells), which could necessitate fast restart of transcription initiation upon mitotic exit. Unfortunately, we have not been able to test our hypotheses on retained mitotic CTCF binding human embryonic stem cells due to our inability to obtain pure populations of living prometaphase-arrested human stem cells. We can, therefore, not conclude whether retained CTCF binding in mitotic pluripotent cells is a common feature across species or unique to mouse pluripotent cell lines. Although we can only speculate about the potential function of maintained CTCF binding upon G1 entry, we did not observe any function related to mitotic chromosome folding by bound CTCF during mitosis. When representing Hi-C data as individual loci or as pile-ups of Hi-C signal on pair-wise interactions of CTCF sites, we did not find any evidence of TADs, insulation boundaries, or CTCF loops despite maintained CTCF binding in mitosis. This suggests that mitotic loop extruding complexes condensin I and II are not blocked by CTCF, in contrast to its interphase counterpart—loop extruding cohesin. Although a CTCF interacting-motif has been described for cohesin (Zhang et al. 2023), it is, to our knowledge, not known whether this motif is present at other SMC complexes such as condensin. Furthermore, a second CTCF-interacting motif to cohesin was recently described (Barth et al. 2025), suggesting a more complex logic mediating the interaction between CTCF and SMC complexes.

Analyzing the average loop length in mitotic mouse cells, we noted a much longer length compared to previous studies with human samples. Indeed, analyzing mouse, human, and chicken data, we could robustly identify species-specific differences in the average length of mitotic loops. It has been shown that mitotic loop arrays are formed by the combined action of condensin I and II, where condensin II mediates loop formation in large loops, with several smaller loops inside formed by condensin I (Gibcus et al. 2018). Additionally, the ratio of condensin I and II modulates the level of condensation and the average loop sizes, as has been observed as cells progress from prophase to mitosis (Gibcus et al. 2018), during development in mitotic *Xenopus* chromosomes (Kieserman and Heald 2011) and when mitotic chromosomes are depleted of either condensin I or II (Shintomi and Hirano 2011). Furthermore, it was shown recently that the *P*(*s*) derivative plots change significantly when condensin I or II are depleted (Gibcus et al. 2018; Samejima et al. 2025). Here, we present additional evidence that when chromosomes have longer arms on average, for example, in mouse as compared to chicken, sister chromatids compact to a greater extent due to the formation of larger mitotic loops. Interestingly, a study by Kramer et al. (2021) provides good confirmations of our proposal: these authors find that the width of the crosssection of mitotic chromosomes scales with the linear length of the genome. Given that the crosssection should be correlated with loop size (Samejima et al. 2025), this suggests that organisms with larger genomes tend to build mitotic loop arrays with larger loops. This process can possibly be mediated by loading different ratios of condensin I and II on mitotic chromosomes or different absolute levels of condensin (Choppakatla et al. 2021; Zhou et al. 2023). Although it has been described that vertebrate species appear to have different ratios of condensin I and II (Ono et al. 2003; Hirota et al. 2004; Ohta et al. 2010; Green et al. 2012; Vagnarelli 2012), to our knowledge, this has not yet been systematically studied in relation to mitotic loop size and chromosome dimensions, with the exception of recent reports in budding yeast and the *Xenopus* embryo (Kakui et al. 2022; Zhou et al. 2023). It would be interesting to support our findings with experimental data capturing ratios of condensin I and II across different species. Although we assume that the condensin machinery is agnostic to which chromosome they are loaded onto and are compacting, we hypothesize that the species-specific loop length is the result of the general condensin loading density and processivity, which have both evolved in each species to ensure sufficient compaction of the longest q-arm length. As a result, loop sizes are similar along all chromosome arms in a given species, regardless of their length (Supplemental Fig. S2A). Although all vertebrate mitotic chromosomes are folded as an array of loops mediated by condensin I and II, the ratio and absolute levels at which condensins are loaded onto chromosomes could modulate the dimensions of chromosomes to generate long and thin or short and wide chromosomes.

## Methods

### Cell culture and synchronization conditions

Mouse embryonic stem cells (E14TG2a) were cultured and synchronized with a 6-h nocodazole arrest following previous publications (Festuccia et al. 2016, 2019). HeLa and C2C12 cells were cultured in DMEM media supplemented with Glutamax-I, 10% heat-inactivated FBS, and penicillin-streptomycin. C2C12 cells were synchronized with nocodazole arrest (50 ng/mL) for 8 h. Mitotic C2C12 and mES cells were harvested by mitotic shake off. Both mitotic and asynchronous cultures were fixed with 1% formaldehyde and stored at −80°C until processed for Hi-C.

### HeLa mitotic synchronization with different lengths of time in nocodazole

HeLa cells were synchronized in G2 for 24 h in 9 µM RO-3306 (Adipogen, AG-CR1-3515-M005). RO-3306 was washed out and replaced with fresh DMEM with 100 ng/mL nocodazole (Sigma, M1404). Floating (mitotic) cells were collected by shake off after 2, 4, or 8 h in nocodazole. Cells were fixed with 1% formaldehyde and stored at −80°C until FACS sorting for mitotic cells.

### C2C12 and HeLa mitotic cell sorting

For mitotic sorting, flash-frozen formaldehyde-fixed cell pellets were thawed on ice and then partially permeabilized on ice for 15 min using 1× PBS (diluted from Gibco, 70013-32),+3% BSA (Sigma, A7906), and 0.1% Saponin (Sigma-Aldrich, 47036-50G-F). Cells were centrifuged for 5 min at 500*g*, supernatant was removed, and cells were resuspended in mouse monoclonal H3 phospho S10 antibody (abcam, ab14955; 1:500) and anti-mouse-Alexa Fluor 405 (abcam, ab175660; 1:1000) diluted in 1× PBS + 3% BSA at room temperature for 90 min. Cells were centrifuged for 5 min at 500*g*, supernatant was removed, washed once with 1× PBS +3% BSA, centrifuged for 5 min at 500*g*, supernatant was removed, and cells were resuspended in 100 µg/mL RNase A (conc, Roche, 10109169001) and 50 µg/mL propidium iodide (Thermo, P1304MP) in 1× PBS for 30 min at room temperature. Cells were sorted for G2/M and H3 phospho S10 staining into PBS +3% BSA using a BD FACS Melody with the following channels: 405 nm laser, 448/45 bandpass filter; 488 nm laser, 488/15 bandpass filter; 561 nm laser, 605LP dichroic mirror, 613/18 filter. After sorting, cells were pelleted by centrifugation, flash-frozen in liquid N2, and stored at −80°C until Hi-C library preparation.

### ATAC-seq

C2C12 cells were cultured as above and arrested in prometaphase using 100 ng/mL nocodazole for 12 h, and mitotic cells harvested by shake-off. The purity of the preparations was assessed by DAPI staining and microscopy and shown to contain 5% of remnant interphase cells. Chromatin accessibility was probed using an adaptation of the ATAC-seq protocol (Buenrostro et al. 2015). Briefly, 100,000 cells were harvested and washed with PBS. Instead of using lysis buffer to isolate nuclei, cells were pelleted by centrifugation for 5 min at 500*g* at 4°C, resuspended in 50 µL of transposition reaction mix (25 µL of Tagmentation DNA buffer, 2.5 µL Tagment DNA enzyme [Illumina Tagment DNA TDE1 Enzyme and Buffer kits, Cat# 20034197], and 22.5 µL nuclease-free H_2_O), and incubated for 30 min at 37°C with gentle agitation. Reactions were stopped by adding the appropriate volume of Binding Buffer (Qiagen MinElute PCR kit), and the DNA was purified using the Qiagen MinElute PCR kit according to the manufacturer's protocol. The purified DNA, eluted in 10 µL, was either stored at −20°C or used directly for library preparation. ATAC-seq libraries were generated using 10 µL transposed DNA, custom made Illumina barcodes previously described (Buenrostro et al. 2013), and KAPA HiFi HotStart (KapaBiosystems, KM2602) for PCR amplification. The number of PCR cycles for PCR amplification was determined using qPCR. Following PCR amplification, libraries were purified using SPRI beads, using a sample to bead ratio of 1:1.4. Concentration and fragment size distribution was determined using an Agilent 2200 Tapestation. ATAC-seq libraries were paired-end sequenced on Illumina NextSeq 500 using 75-bp paired-end reads in biological duplicates.

### ATAC-seq analysis

ATAC-seq sequencing reads were trimmed to 24 bp and aligned to reference genome mm10 using Bowtie 2 with a maximum mapping length of 2000 bp (Langmead and Salzberg 2012; Buenrostro et al. 2013). Paired-end reads were filtered for mapping quality, mitochondrial reads, and PCR duplicates. mESC ATAC-seq data were plotted as V-plots (Zentner and Henikoff 2012) on all interphase bound CTCF motifs (51,805 sites) and on CTCF motifs categorized as bookmarked (10,799 sites), reduced (18,704 sites), or lost (22,302 sites) in mitosis as characterized by Owens et al. (2019). V-plots were produced as described (Oomen et al. 2019). To plot V-plots, CTCF motifs were oriented in the same direction. C2C12 ATAC-seq data were analyzed and processed as described in Oomen et al. (2019). Interphase and mitotic bound CTCF sites were identified when a peak in ATAC-seq data overlapped with a CTCF motif in interphase and/or mitosis.

### Hi-C

Hi-C on mitotic and asynchronous cultures was performed according to a previously published protocol (Belaghzal et al. 2017). Briefly, cells were fixed and stored as described above. Crosslinked cells were thawed, lysed, and digested with DpnII restriction enzyme overnight at 37°C. Restriction overhangs were filled with biotin-14-dATP supplemented with dTTP, dCTP, and dGTP for 4 h at 23°C, followed by ligation using T4 DNA ligase at 16°C for another 4 h. Samples were then treated with Proteinase K at 65°C overnight. DNA was cleaned up and purified using phenol:chloroform and ethanol precipitation. DNA was sonicated and size-selected to an average size of 100–350 bp using AMpure XB beads, followed by end repair. Samples were enriched for biotin-tagged DNA fragments by pull down using streptavidin beads. After A-tailing, libraries were ligated with indexed Illumina TruSeq sequencing adapters, followed by PCR amplification. Finally, libraries were cleaned up from PCR primers using Ampure XP beads and sequenced using paired-end 50-bp sequencing on an Illumina HiSeq 4000 or NextSeq 2000.

### Hi-C mapping and downstream analysis

Hi-C sequencing files were mapped to reference genomes hg38 (HeLa data), galGal7 (DT40 data), and mm10 (C2C12, mESC, and C127 data) using the publicly available distiller-nf mapping pipeline (https://github.com/mirnylab/distiller-nf) and downstream analysis tools pairtools (https://github.com/mirnylab/pairtools), cooltools (https://github.com/mirnylab/cooltools), and the open2c tool suite (https://open2c.github.io/). For DT40 data, processed data as mcool files (mapped to and galGal7) were downloaded directly from the NCBI Gene Expression Omnibus (GEO; https://www.ncbi.nlm.nih.gov/geo/) under accession number GSE262525. Briefly, reads were mapped using BWA-MEM, PCR duplicates were removed, and reads were filtered for mapping quality. Distance decay and derivative plots were created using cooltools code by calculating contact frequency (*P*) as a function of genomic distance (*s*) using mcool files. For further downstream analysis, interactions were binned in matrices at a range of different resolutions using cooler (Abdennur and Mirny 2020). Iterative balancing was applied to all matrices, while ignoring the first two bins from the diagonal (Imakaev et al. 2012). Pile-up plots at single CTCF sites and pairwise CTCF interactions were produced using observed over expected signal binned at 10 kb. Pairwise CTCF sites for pile-up plots were predicted by pairing all CTCF sites within 250 kb on the same chromosome within the CTCF category (CTCF sites bookmarked in mitosis, reduced in mitosis, or lost in mitosis) as curated by Owens et al. (2019). Directionality of the CTCF motifs were taken into account, and all motifs were orientated in the same direction.

### Mitotic chromosome spreads and chromosome labeling for imaging

Asynchronous HeLa or C2C12 cultures were incubated in 0.1 µg/mL colcemid (Sigma-Aldrich, 10295892001) for 2 h. Both cell lines were processed in the same way, as follows; cells were collected after trypsinization, spun down at 4°C at 1000*g* for 10 min, and all but 500 µL media removed. Cells were then resuspended in the remaining media, and 5 mL prewarmed (37°C) 75 mM KCl were added dropwise. Cells were swollen at 37°C for 10 min, then fixed in freshly made ice cold 3:1 methanol acetic acid. Aliquots of the fixed samples were then dropped onto slides, and the slides set, chromosome side up, over a beaker with 70°C–80°C distilled water for 30 sec. Slides were then air-dried and incubated at 37°C overnight prior to using for DNA-FISH experiments. To identify HeLa S3 Chromosome 14 and C2C12 Chromosome 18, custom Atto 565-labeled MyTags libraries (Arbor Biosciences/Daicel) were used to stain mitotic chromosomes spreads (HeLa—Chr 14: 100,674,834–100,852,919; C2C12—Chr 18: 88,639,179–88,816,381). Centromeres were labeled with the pan-centromeric probe CENPB-Cy5 (PNA Bio, F3005). After DNA FISH and CENP-B probe labeling, slides were stained in 300 nM 4′,6-diamidino-2-phenylindole (DAPI, Thermo Fisher Scientific, D1306) and mounted in ProLong Diamond antifade mountant (Invitrogen, P36965).

### Confocal fluorescence imaging

Confocal images were acquired on a Leica SP8 spectral confocal microscope (housed in UMass Chan's Sanderson Center for Optical Experimentation, SCOPE; RRID: SCR_022721) equipped with a 63×/1.40 NA PL Apo CS2 oil immersion lens (Leica); 405 nM and 638 nM Diode lasers and 552 nM OPSL laser; and sCMOS cameras (pco.edge). For HeLa chromosomes, the spectral detector settings used were PMT 410 nm–560 nm (405 laser), HyD2 560–633 nm (552 laser), and HyD3 643–783 (638 laser). For C2C12 chromosomes, the spectral detector settings used were PMT 410 nm–575 nm (405 laser), HyD2 557–778 nm (552 laser), and HyD3 643–783 (638 laser). Pixel size was 24 nm, frame size was 1024 × 1024, and zoom was 7.6×. Image stacks with 0.3-µm-thick z sections were acquired using immersion oil with a refractive index of 1.518. After image acquisition, Lightening deconvolution was applied to each image stack.

### Image analysis

Chromatid length was measured using Fiji (Schindelin et al. 2012). Image stacks were projected into maximum intensity Z-projections. HeLa Chromosome 14 and C2C12 Chromosome 18 were identified by FISH DNA probe staining, and one sister chromatid was measured for length (from the end of the arm to the beginning of the centromere stained by CENPB). Fifty C2C12 chromatids and 49 HeLa chromatids were measured. Length measurements were analyzed in GraphPad Prism 9.5.1, using an unpaired *t*-test.

### Publicly available data used in this study

In addition to the Hi-C data that were generated for this study, we use several ATAC-seq and Hi-C data sets that are publicly available on the NCBI Gene Expression Omnibus (GEO). ATAC-seq data in mESC (Festuccia et al. 2019) are available under accession number GSE122589. Hi-C HindIII data in mitotic HeLa are available under GSE102740 (Gibcus et al. 2018), and GSE133462 for DpnII Hi-C data (Abramo et al. 2019). Hi-C data of G2 synchronized and mitotically synchronized (60 min timepoint) can be found in GSE262525 (Samejima et al. 2025). Hi-C data of mouse cell line C127 can be found under GSE149677 (Fitz-James et al. 2020).

### Software used in this study

Hi-C mapping pipeline distiller-nf is available on GitHub (https://github.com/mirnylab/distiller-nf). Downstream analysis tools pairtools and cooltools are available through GitHub (https://github.com/mirnylab/pairtools, https://github.com/mirnylab/cooltools, and https://open2c.github.io/). Code used for analysis of ATAC-seq data can be found at GitHub (https://github.com/dekkerlab/CTCF_in_mitosis_GR_2018).

## Data access

All raw and processed sequencing data generated in this study have been submitted to the NCBI Gene Expression Omnibus (GEO; https://www.ncbi.nlm.nih.gov/geo/) under accession number GSE249331. All microscopy data have been submitted to the BioStudies database (https://www.ebi.ac.uk/biostudies/) under accession number S-BIAD953.

## Supplemental Material

Supplement 1
