# Supplementary material for: Mitotic chromosomes harbor cell type– and species-specific structural features within a universal loop array conformation
Source: Genome Res. 2025 Aug;35(8):1733–44. doi: 10.1101/gr.280648.125 (PMC12315709; doi:10.1101/gr.280648.125)
Supplement: Supplement 1 [file Supplemental_Materials.pdf]

Supplemental Materials – Table of Contents

Supplementary Figures

Figure S1 - ATAC-seq data obtained with mESCs show that a set of CTCF motifs remain bound by CTCF in mitosis, whereas other CTCF motifs lose binding

Figure S2 - Mitotic loop arrays species for chromosomes investigated by Hi-C

Figure S3 – Q-arm compaction as measured by microscopy

Supplemental Figure S1

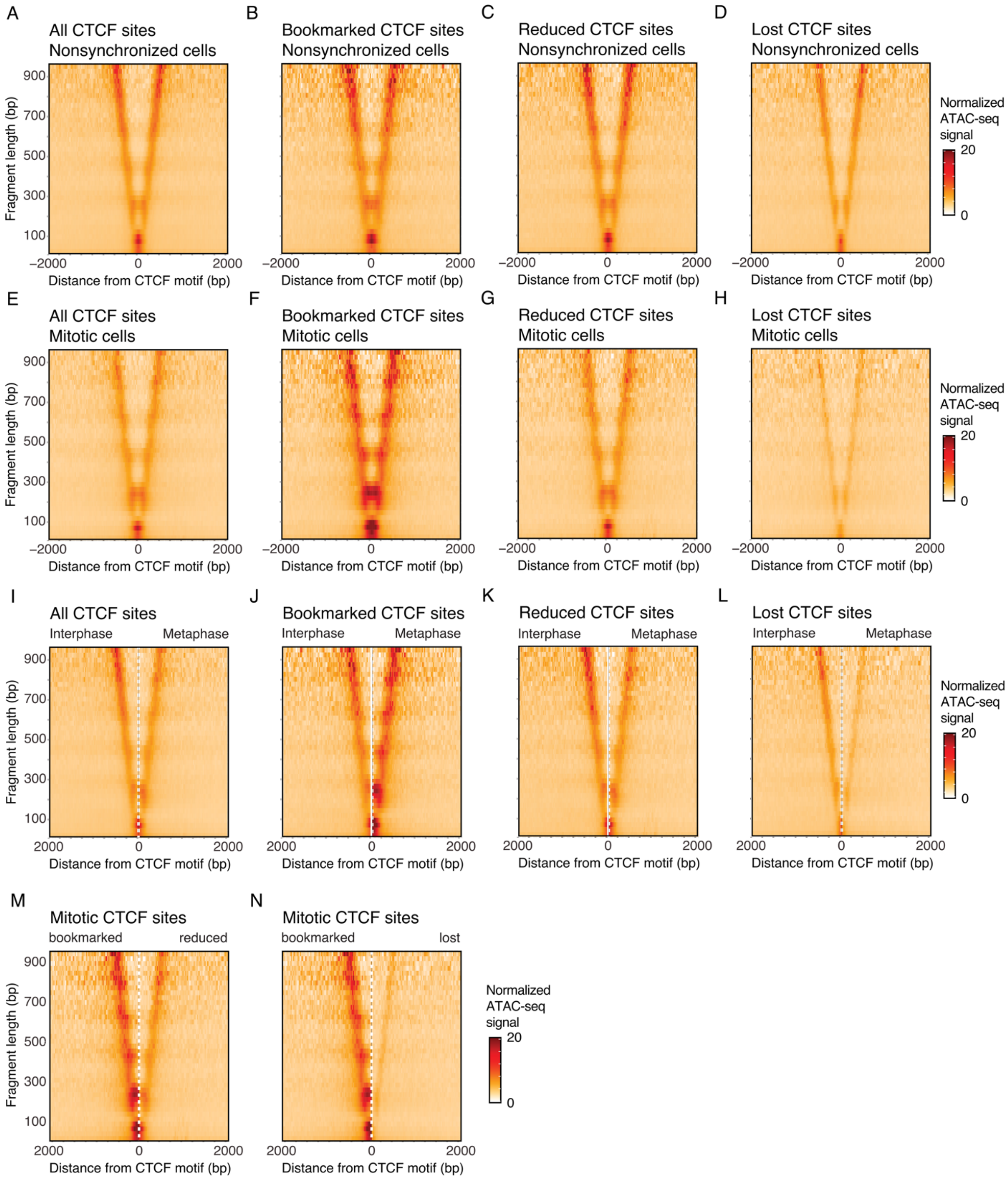

Figure S1 – ATAC-seq data obtained with mESCs show that a set of CTCF motifs remain bound by CTCF in mitosis, whereas other CTCF motifs lose binding. (**A-D**) ATAC-seq data of nonsynchronized mESCs represented in V-plots as a pile up on all interphase-bound CTCF sites (51,805 sites total) (A),

bookmarked CTCF sites (10,799 sites) (B), CTCF sites with reduced CTCF binding (18,704 sites) (C) and CTCF sites that lose CTCF binding in mitosis (22,302 sites) (D). (**E-H**) ATAC-seq data of mESCs synchronized in mitosis represented in V-plots as a pile up on all interphase-bound CTCF sites (E), bookmarked CTCF sites (F), CTCF sites with reduced CTCF binding (G) and CTCF sites that lose binding in mitosis (H). (**I-L**) Side-by-side comparison of V-plots for nonsynchronized and mitotically synchronized cells on all interphase-bound CTCF sites (I), bookmarked CTCF sites (J), reduced CTCF sites (K) and CTCF sites that lose binding in mitosis (L). (**M-N**) Side-by-side comparison of V-plots of mitotically synchronized mESCs on bookmarked CTCF sites vs reduced CTCF sites (M) and bookmarked CTCF sites vs lost CTCF sites (N).

Supplemental Figure S2

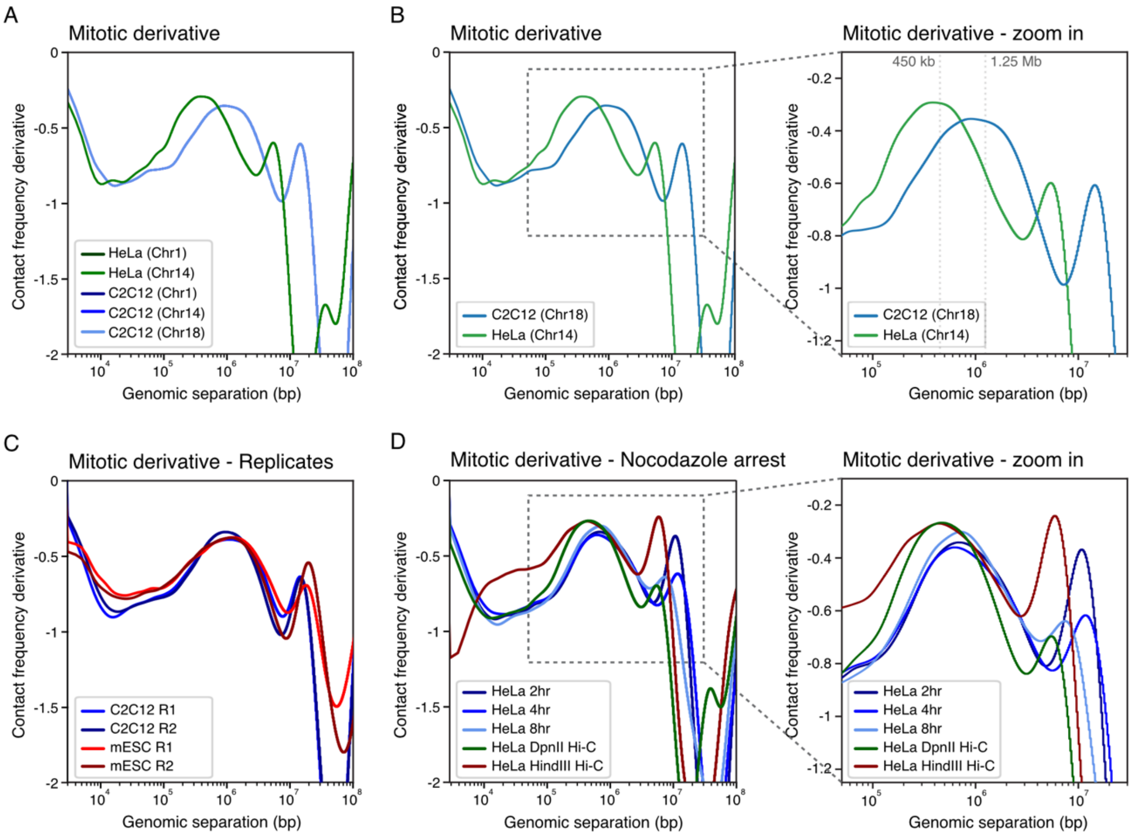

Figure S2 – Mitotic loop arrays species for chromosomes investigated by Hi-C. (**A**) Mitotic derivative plots of different chromosomes in HeLa (Chr1 and Chr14) and C2C12 (Chr1, Chr14 and Chr18) show identical loop sizes across chromosomes. Note that the derivative plots of individual chromosomes are highly similar and therefore indistinguishable as individual lines (**B**) Derivative plots of Hi-C data from HeLa (Chr14) and C2C12 cells (Chr18) synchronized in mitosis, which were investigated by microscopy in figure 4F and a zoom-in of the derivative plot shown in figure 4B, with dashed lines marking the mitotic loop sizes of human and mouse at 450 kb and 1.25 Mb. (**C**) Derivative plots of Hi-C replicates of mitotically arrested C2C12 and mESC. (D) Derivative plots of Hi-C data of HeLa cells performed using different nocodazole arrest timings (in blue coloring) and using different restriction enzymes in the Hi-C protocol (DpnII in dark green and HindIII in dark red).

Supplemental Figure S3

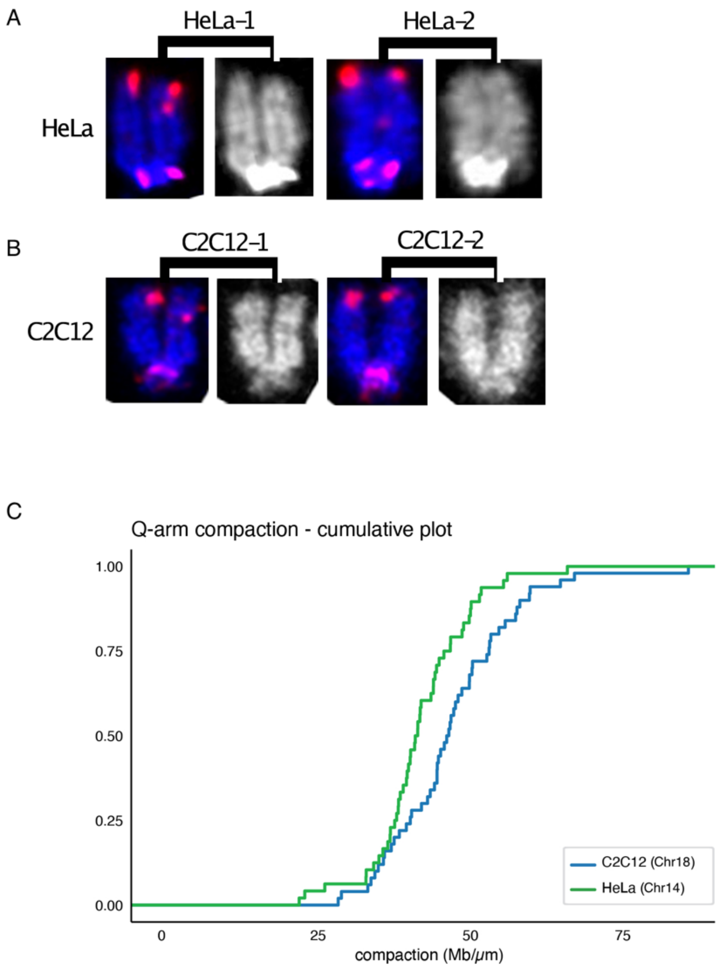

Figure S3 – Q-arm compaction as measured by microscopy. (**A-B**) Representative images of Chr14 in mitotic HeLa cells (A) and Chr18 in mitotic C2C12 cells (B). The left image in each group shows DAPI staining (blue), FISH probe staining (red) to identify the chromosome to measure, and the centromere (magenta). The right image in each group is the DAPI channel in grayscale. Each chromosome was measured in FIJI using the freehand line tool to draw a line between the centromere and the end of the Q-arm. (**C**) Cumulative plot of the Q-arms of mitotic HeLa Chr14 and C2C12 Chr18.
